# Spatial distribution of fat infiltration within the paraspinal muscles: implications for chronic low back pain

**DOI:** 10.1007/s00586-022-07296-7

**Published:** 2022-07-01

**Authors:** Karim Khattab, Lucas K. Dziesinski, Rebecca Crawford, Alex Ballatori, Priya Nyayapati, Roland Krug, Aaron Fields, Conor W. O’Neill, Jeffrey C. Lotz, Jeannie F. Bailey

**Affiliations:** 1 Department of Orthopaedic Surgery, University of California, San Francisco 95 Kirkham St, San Francisco, CA 94122 USA; 2 Body Urbanist, Hünenberg See, Zug, Switzerland; 3 Department of Radiology, University of California, San Francisco, USA

**Keywords:** Fat infiltration, Paraspinal muscles, Multifidus, Chronic low back pain, MRI

## Abstract

**Purpose:**

Fat infiltration (FI) of the paraspinal muscles (PSMs) measured using MRI is an aspect of muscle quality and is considered to be worse in chronic low back pain (cLBP) patients. However, there is not a clear association between paraspinal muscle FI and cLBP, leaving the clinical importance of paraspinal muscle composition unestablished. The spatial distribution of FI in the PSMs may inform mechanistic understanding of non-specific cLBP as it relates to degenerative intervertebral disc (IVD) pathology. We hypothesized that paraspinal muscle fat-mapping would reveal distinct FI distribution patterns in relation to cLBP symptoms and proximity to symptomatic IVD degeneration.

**Methods:**

From advanced-sequence water-fat MRI of 40 axial cLBP patients and 21 controls, we examined the spatial distribution of paraspinal muscle FI in relation to the center of rotation at the L4L5 disc. Using statistical parametric mapping, we compared FI patterns for multifidus (MF), erector spinae (ES), and psoas between patients and controls, and to the presence and severity of adjacent degenerative IVD pathology.

**Results:**

The spatial distribution of PSMs FI differs between PSMs and according to symptoms and the adjacent degenerative IVD pathology. Furthermore, the region of MF closest to the disc center of rotation appears most susceptible to FI in the presence of symptomatic IVD degeneration.

**Conclusion:**

Our study identified spatial distribution patterns of FI in the PSMs as a potential diagnostic biomarker that may also provide granular mechanistic insights into spine biomechanics related to cLBP, as well as advancing the use of prior summary measures limited to overall muscle FI.

**Supplementary Information:**

The online version contains supplementary material available at 10.1007/s00586-022-07296-7.

## Introduction

Chronic low back pain (cLBP), the world's most disabling condition, is the highest (non-cancer) reason for opioid prescription in the USA, and its rates are on the rise with the aging population [1]. While axial cLBP (non-radicular) is commonly considered non-specific and multifactorial in nature, it is often suspected that dysfunction of the spinal stabilization system which includes the intervertebral disc (IVD) and adjacent paraspinal muscles (PSM) plays an important role [2]. Non-specific cLBP is also notoriously difficult to treat because of the uncertainty about causal mechanisms. Dynamic tissues including the PSMs represent a promising rehabilitation target for individualized care. Imaging studies indicate significant association between degenerative IVD pathologies (e.g., disc degeneration, Modic changes, endplate pathology) and pain [3, 4]. However, how these potential pain generating pathologies may lead to disability and poor biomechanical function in cLBP patients is not known. Given the critical role of the paraspinal muscles as a dynamic spinal stabilizer, clarifying the relationship between cLBP symptoms and degenerative IVD pathology on adjacent paraspinal muscle health is a critical step forward to better understand compromised spinal stability and overall biomechanical function with cLBP.

MRI-based characterization of paraspinal muscle composition by quantifying fat infiltration (FI) is a popular approach for assessing muscle quality for spine conditions. However, the relationship between paraspinal muscle FI and cLBP is not straightforward and the underlying mechanistic structure–function relationship remains poorly understood despite advances in quantifying both paraspinal muscle FI and degenerative IVD pathology [5, 6]. Inconsistencies in associations across clinical studies are most notably due to the natural degenerative cascade as a result of aging [7]. However, prior studies of paraspinal muscle FI rely on summary statistics which complicate efforts to tease out the individual contributions of the many factors which may be at play in chronic low back pain. Paraspinal muscle FI is most commonly quantified using conventional summary measurements in which only the overall mean FI% within a muscle cross-sectional area or volumetric region is reported. However, summary measurements for paraspinal muscle FI lack granularity as they do not capture specific locations of fat accumulation in the muscle. This approach is considered the standard for paraspinal muscle FI reporting and results in a loss of information and a lack of sensitivity to spatial patterns of FI accumulation within a given muscle. Given prior observation of structurally and functionally unique regions within individual PSMs, notably the multifidus, we hypothesize that age-related and pain-related factors may differentially affect distinct regions within the PSMs. A spatial approach to characterizing paraspinal muscle FI may identify specific patterns both between and within individual PSMs [8] that better reflect the complex morphology [9, 10] and biomechanical function [11] most notably for multifidus (MF). Furthermore, quantifying the spatial distribution may illuminate underlying mechanisms of FI% by revealing which structural and functional features of the lumbar PSMs increase susceptibility to FI in the presence of pain-generating spinal structures. As such, developing and testing a method for assessing the spatial distribution of FI within the PSMs may result in a more specific, more consistent and more clinically meaningful characterization of paraspinal muscle quality and its underlying mechanisms.

To investigate whether spatial distribution of paraspinal muscle FI relates to symptoms and adjacent degenerative IVD pathology, we generated paraspinal muscle ‘fat-maps’ using axial MRI and referencing the center of rotation (CoR) at the L4L5 disc segment. We hypothesized that paraspinal muscle fat-mapping would reveal distinct FI spatial distribution patterns in relation to cLBP symptoms and proximity to the CoR of a symptomatic IVD degeneration. Due to its key role in spinal segment stabilization, we expected that the region of multifidus closest to the CoR would display the highest fat content adjacent to symptomatic IVD degeneration.

## Methods

### Sample

With IRB approval (IRB#13–12,224; approved August 21, 2014) and informed consent, 3 T multi-echo MRI was collected for 40 cLBP patients and 21 age- and sex-matched controls. Patients with more than three months of LBP (VAS ≥ 4 or ODI ≥ 30) and between ages 18 and 70 were included. Exclusion criteria for enrollment included pregnancy, diabetes, smoking, cancer, spondylolisthesis, scoliosis, prior lumbar surgery, disc herniation, compression fractures, taking osteoporosis medication. Controls reported no prior history of back pain (VAS ≤ 1) or known spinal conditions. VAS scores were collected once, at baseline, directly prior to the subject’s MRI visit.

### MRI scans, pathology scoring, and muscle segmentation

Lumbar scans were performed on a 3 T scanner and sequences included standard clinical T1- and T2- weighted MRI sequences and advanced-sequences used for cartilage endplate detection (high-resolution 3D ultrashort echo time (UTE) [12]), disc composition (combined T_1ρ_ and T_2_ mapping [13]), and FI (iterative decomposition of water and fat with echo asymmetry and least-squares estimation (IDEAL) [14]). Specifications referenced in prior publications ([13–15]). Cartilage endplate pathology (CEP) was measured based on the presence and absence of defects in the cartilage using UTE [14]. Modic changes (MC) were graded by experienced clinicians using T1 and T2 standard clinical sequences. Disc degeneration was scored using Pfirrmann grade (PG). The UTE sequence specifications included TE = 0.075 ms, TR = 10 ms, voxel size of 0.22 × 0.22 × 0.80 mm3, and fat suppression. The IDEAL sequence specifications included TR = 7 ms, TE = 2.1 ms, flip angle = 3°, rBW =  ± 83.3 kHz, FoV = 22 cm, in-plane resolution = 1.3 mm, and slice thickness = 4 mm). Multifidus, erector spinae, and psoas were manually segmented from a combination of T1- and T2-axial images, then segmentations were validated and transferred to IDEAL images for fat-mapping (intra-rater ICC: 0.98, *P < *0.001; inter-rater ICC: 0.99, *P < *0.001; see [14] for full description of muscle segmentation).

### Spatial distribution of FI% within PSMs (fat-maps)

**Fig. 1:**
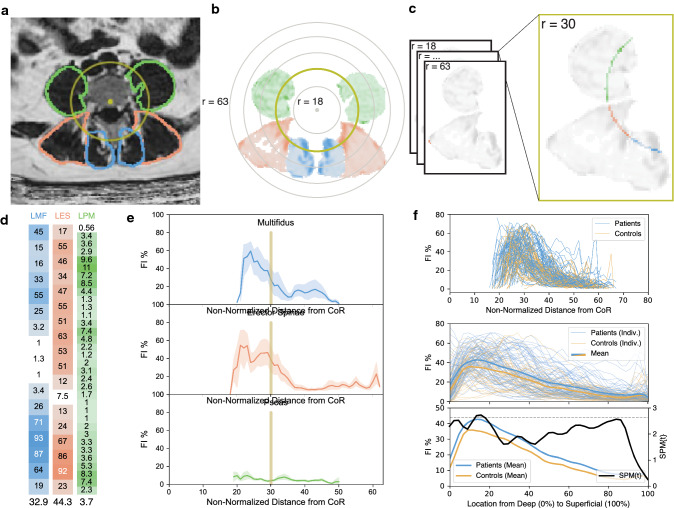
Workflow from MRI segmentation to statistical parametric mapping analysis (SPM) for paraspinal muscle fat maps **A**) Location of center of rotation (CoR) used to center the radial ROIs was calculated and identified. **B** CoR coordinates were applied to matrix with paraspinal muscle segmentation. **C** FI% pixels within each ROI overlaying separate muscles were collected and transformed to columns (**D**) for each radial ROI per muscle. Each column entry represents the FI% of a single pixel. **E** For each radial ROI, FI pixels were averaged and the mean FI at every ROI was plotted for each muscle. **F** The radial fat distribution for separate muscles was graphed for all subjects (top) and then normalized based on individual radial muscle width so that the x-axis ranged from 0 to 100% of the radial width (middle). The mean distribution was then calculated for each subgroup. Then, regional differences in FI between groups were identified using SPM t-tests (bottom). Differences in FI were deemed significant between groups if the SPM{t} for a given region surpassed the critical threshold line (dotted line). As shown, multifidus FI was significantly larger in patients compared to controls in the 13.4–17.2% range of the distribution (*p = *0.044) where the SPM{t} statistic surpassed the critical threshold (t* = 2.643)

Our novel fat-mapping method is depicted in Fig. 1. At the center of L4L5 disc segment for each subject, the coordinates for the motion segment CoR were approximated at the center point within the posterior quarter of the mid-sagittal AP diameter of the IVD (12.5% from posterior edge of disc) [16]. We defined multiple circular regions of interest (ROI) radiating outward from the CoR at increments of one pixel and calculated the average for each radial ROI. The method recognizes that each muscle has a unique range and number of non-overlapping ROIs based on size and radial distance from the CoR. The mean FI% among all pixels in each radial ROI from individual muscles was calculated and plotted, with a three-point moving average, as a nonlinear fat distribution curve depicting the spatial variability in a muscle’s FI% moving away from the CoR. Each curve was then distance normalized so that the x-axis ranged from 0 to 100% of the radial muscle width. Fat-maps were created using Python (Python Software Foundation, www.python.org).

### Statistical data analysis

The mean FI% of the whole muscle (overall mean FI%) and the fat-maps were separately compared between patient and control groups and further stratified based on spinal pathology measurements from the adjacent disc segment. Statistical analyses utilized unpaired t-tests (depending on groups and conditions compared) to compare overall mean FI% values. For the fat-maps, we utilized statistical parametric mapping (SPM) to conduct statistical analyses across the entire fat-map. In prior studies, SPM has been used to analyze regional differences in intramuscular damage within the quadriceps femoris [17]. We calculated the SPM t-test t-statistic (SPM{t}) at every point from 0–100% of the fat-maps. Then, we calculated the critical threshold t-statistic (α = 0.05) so that any region of the fat-map where the SPM{t} statistic surpassed the critical threshold was deemed significant for a given analysis. The size of these significant regions (clusters) correlates with the strength of significance. All SPM analysis was performed using the SPM1D package in Matlab.

## Results

**Fig. 2:**
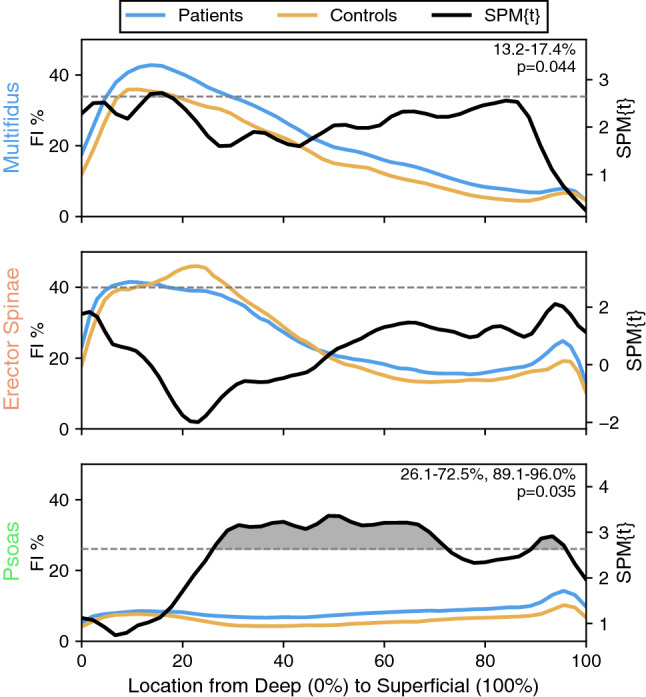
Differences in FI distribution patterns between cLBP patients and controls are muscle-specific and unique across PSMs Comparison of paraspinal muscle fat-maps between cLBP patients and controls using SPM t-tests reveal localized differences in FI in the deep multifidus (top) and in the intermediate to superficial psoas (bottom) but not in the erector spinae (middle). Although changes in the multifidus and the psoas FI distributions are associated with the presence of cLBP symptoms, the associated differences in FI are in separate regions of the muscle. Differences in FI were deemed significant between groups if the SPM{t} for a given region surpassed the critical threshold (dashed black line). Regions of significance (clusters) are shaded for visualization. The shaded area of a given cluster is associated with the strength of significance

Mean age and BMI did not significantly differ between cLBP patients (48.8 ± 12.2 years, 25.6 ± 5.1 kg/m^2^) and controls (43.5 ± 12.7 years, 23.8 ± 4.1 kg/m^2^). The percent representation of females to males was 50.0% for the cLBP patients and 47.1% for the control group. There were no significant differences in either age or BMI across any two groups separated for statistical analysis. The overall mean FI% in the multifidus and psoas were significantly greater in patients compared to controls (multifidus: 4.85%, *p = *0.005; psoas: 2.29%, *p = *0.003; Supplemental Table 1). Similarly, SPM identified specific regions with higher FI% in patients versus controls, specifically in the deep multifidus and intermediate to superficial psoas (*p < *0.05–*p < *0.001; Fig. 2; Supplemental Table 2–4). For the erector spinae, both the overall mean and spatial distribution of FI% was not different between patients and controls.

### Paraspinal muscle FI and its association with degenerative IVD pathology

**Fig. 3:**
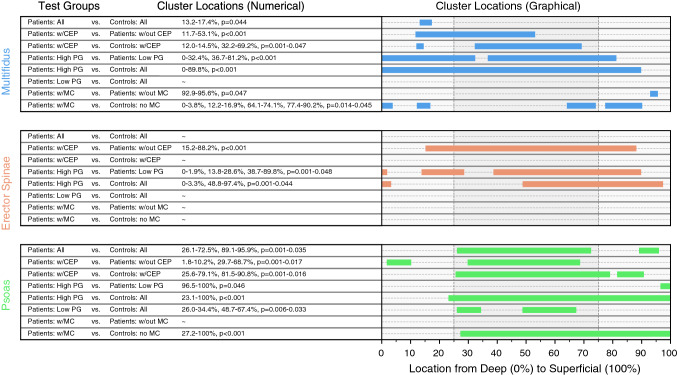
Cluster locations derived from SPM show muscle specific FI distribution patterns in relation to presence and severity of degenerative IVD pathology SPM t-tests were conducted to compare FI distributions between subgroups of patients and controls stratified by: the presence of CEP damage, high (> 3) or low ( ≤ 3) Pfirrmann grade, and the presence of MC. For each analysis, the locations of the regions of significance (clusters) were identified using SPM t-tests and are listed numerically and shown graphically to depict muscle-specific trends. Each cluster location represents the region within the muscle where there is a significant difference in fat infiltration found between groups. In the multifidus fat-maps (top), significance clusters are most present in the deep to intermediate regions of the muscle, while, in the erector spinae and psoas fat-maps, significance clusters are most present in the intermediate to superficial regions of the muscle. Spatial patterns of FI in the PSMs are thus associated with the presence and severity of degenerative IVD pathology. Associated differences in FI are localized in unique regions of each paraspinal muscle

**Fig. 4:**
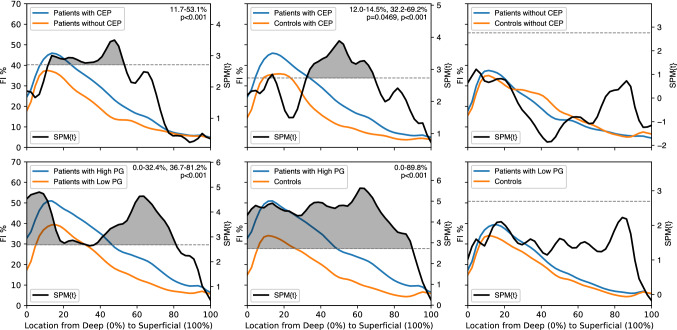
Analysis of multifidus fat maps using SPM t-tests reveal the individual and interactive associations of pain and pathology with spatial FI distributions Top row: Analysis of patient subgroups stratified by the presence of CEP damage (left) shows significantly greater FI% in regions of the multifidus in patients with CEP compared to patients without CEP. Additionally, comparing patients with CEP damage to controls with CEP damage (middle) reveals significantly larger FI% for patients in regions of the multifidus which are not significant in the original, unstratified patients vs. controls assessment (Fig. 2, top). Lastly, the multifidus fat-maps for patients without CEP did not differ from controls without CEP. Bottom row: Similar to CEP, stratifying patients based on high (> 3) and low(< = 3) PG revealed significant differences in FI distribution. Patients with high PG had significantly greater FI compared to patients with low PG (left) and controls (middle). However, there were no significant differences in the FI% in any region of the multifidus when comparing patients and controls with low PG (right). These results show that the presence of degenerative IVD pathology is associated with spatial differences in multifidus FI within patients, but the presence of symptoms in the absence of pathology is not a distinguishing factor in the spatial distribution of multifidus FI

While there was a significant difference between patients and controls in both the overall FI% and its distribution, these differences were greater when degenerative IVD pathology was present. In the multifidus and psoas, overall mean FI% differed significantly between groups stratified by both symptoms and degenerative IVD pathology. There was also a significant difference in FI% distribution related to symptoms and degenerative IVD pathology (CEP, PG, MC) concentrated in the deep to intermediate regions of the multifidus (*p < *0.05–*p < *0.001; Figs. 3 and 4). For erector spinae, there were significant spatial distribution differences in FI% related to degenerative IVD pathology (CEP, PG) concentrated in the intermediate to superficial regions (*p < *0.05–*p < *0.001; Fig. 3). For psoas, there were significant spatial distribution differences in FI% related to symptoms and degenerative IVD pathology (CEP, PG, MC), with FI concentrated in the intermediate to superficial regions (*p < *0.05–*p < *0.001; Fig. 3). For patients with CEP and high PG, paraspinal muscle fat-maps showed a significantly larger FI within specific regions compared to patients without these pathologies. Disc composition was not associated with differences between patients and controls in the fat-maps for any of the PSMs.

### Shape of FI distribution related to patient-reported pain

**Fig. 5:**
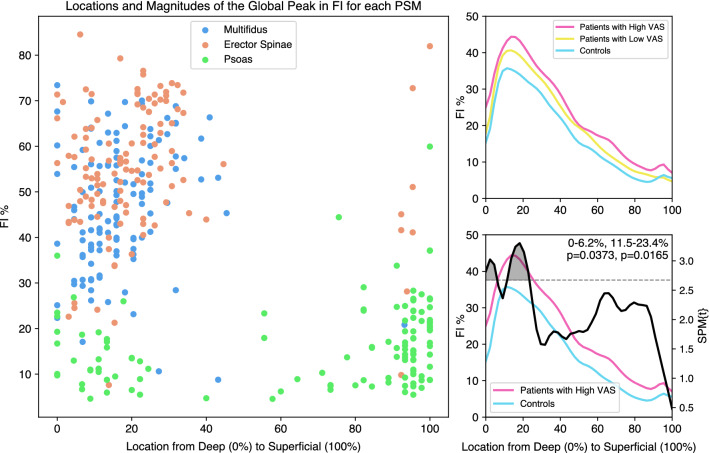
Stratifying subjects into three groups using subject reported pain severity (VAS) shows a shift in multifidus FI between groups, focused near the peak of the FI distribution in the deep multifidus Left: The location of the multifidus and erector spinae global peak in FI consistently falls in the deep region of the muscle, whereas there is more variability in peak location in the psoas. Only the multifidus global peak magnitude is significantly different between patients and controls. Right: SPM analysis shows significant differences in FI distribution at the global peak in multifidus FI between patients with high VAS and controls (bottom). Patients with low VAS scores were not different from controls, showing a pain-associated shift in FI localized at the deep multifidus

The percent of individual muscles within our sample that had their highest peak of FI in the deep region was 99.2% for multifidus, 96.7% for erector spinae, and 33.6% for psoas (Fig. 5). The highest peak in FI for multifidus was 6.5 percent points higher for patients (49.3% ± 12.8% at 16.5% ± 9.9% radial depth) compared to controls (42.8% ± 14.8% at 18.1% ± 15.8% radial depth; *p = *0.013). The highest peak in FI for psoas was 4.6 percent points higher for patients (17.9% ± 9.1% at 73.9% ± 35.1% radial depth) compared to controls (13.3% ± 7.5% at 57.6% ± 40.2% radial depth; *p < *0.01). There was no significant difference in peak FI for erector spinae between patients (55.5% ± 13.0% at 23.2% ± 23.0% radial depth) and controls (53.7% ± 16.9% at 21.6% ± 18.0% radial depth).

When comparing fat-maps for PSMs between high (> 6) and low (≤ 6) patient-reported pain (VAS), we observed trends between fat-map patterns progressing from controls with no pain to cLBP patients with high VAS (Fig. 5). For multifidus, fat-map patterns for high VAS were significantly different from controls (0–6% & 12–23%, *p = *0.02–0.04), but low VAS was not different from either high VAS or controls. There were no differences in fat-maps for high and low VAS with controls for erector spinae or psoas.

## Discussion

Using a novel application of fat-mapping analysis, we identified different spatial distribution patterns of FI% between and within the PSMs and in relation to symptoms and degenerative IVD pathology. First, with respect to cLBP symptoms alone, we identified differences in whole muscle and spatial distribution of FI% for multifidus and psoas, with different PSMs showing distinct regions of peak FI% in regard to the IVD CoR. Second, when examining differences based on degenerative IVD pathology, we found that cLBP patients with CEP damage and higher IVD degeneration (PG) had larger regions of significantly higher paraspinal muscle FI% compared to patients without these pathologies. Furthermore, fat-maps from cLBP patients with low IVD degeneration and no CEP damage did not differ from controls. Lastly, the shape of the spatial distribution of FI% relative to the IVD showed a significantly higher peak of FI in multifidus between patients and controls located in the deepest part of the muscle closest the CoR. When comparing the shape of the fat-maps between patients with relatively high and low self-reported pain, we identified a progression of increasing FI% specifically in the deep region of multifidus. Our results suggest that 1) spatial distribution patterns of FI in the PSMs differ with respect to symptoms and adjacent degenerative IVD pathology and 2) that the deep region of the multifidus has the highest amount of FI in relation to symptomatic IVD degeneration.

Prior studies seeking to associate paraspinal muscle FI with cLBP have shown an inconsistent relationship between FI and patient-reported symptoms or outcomes [5]. This may be due to a lack of specificity in reporting an overall mean FI% [8, 18]. While we showed general agreement between the paraspinal muscle fat-maps and the overall mean FI% between patients and controls, our fat-mapping method further identified relevant mechanistic associations between pain measures and paraspinal muscle quality. Generating these fat-maps enabled us to identify distinct spatial distribution patterns of elevated paraspinal muscle FI in association with cLBP symptoms and the presence of degenerative IVD pathology. Our study specifically recruited patients with axial cLBP to investigate the PSMs relationships with symptomatic IVD degeneration. We found that the presence of CEP, high IVD degeneration (PG), and Modic changes related to larger distinct regions of higher FI in adjacent PSMs, most pronounced in the deep region of multifidus. Further, both higher levels of self-reported pain and higher IVD degeneration associated with higher FI in the deep multifidus, suggesting that as cLBP symptoms and IVD degeneration progresses there may be more marked changes to paraspinal muscle FI. Therefore, paraspinal muscle fat-maps may serve as a novel imaging-based biomarker that relates to the adjacent degenerative IVD pathology.

It is unclear how or why PSMs become infiltrated with fat in cLBP patients; however, changes in loading on different muscle regions or fascicles may introduce susceptibility to FI. Muscular dystrophy patients show predominant accrual of FI in muscle attachment sites. In the rotator cuff, unloaded muscle tissue post tear has higher FI [19]. Our findings of increased FI in the deep region of the multifidus in relation to symptoms and IVD may be a consequence of arthrogenic muscle inhibition [20]. The reduced activity of the shorter (deep) multifidus fascicles in response to a dysfunctional motion segment through pain (symptomatic IVD) or altered loading (passive structure laxity) and mechanical insufficiency of shorter fascicles more intimate to the CoR [10, 21, 22] may lead to muscle atrophy and FI. This is supported by prior findings of reduced electromyography amplitude in deepest multifidus fascicles in patients with a history of LBP [23]. While a clear explanation remains elusive, our results appear to support a compensatory (delayed) biomechanical response to IVD changes from the PSMs and particularly of multifidus fascicles closest to the IVD [7].

Our results reveal the highest distribution of FI in the deepest multifidus region in relation to a symptomatic IVD degeneration in cLBP patients. Of the structural changes that PSMs are thought to undergo in the transition from acute to chronic LBP, FI arguably occurs slower than atrophy and is therefore considered a characteristic of chronicity [24]. Paraspinal muscle FI is often present with other changes to the muscle including fiber-type changes from fatigue-resistant slow-twitch (Type 1) fibers to fast-twitch (Type 2) [25] and fibrosis [26], which together alter muscle plasticity [11]. Multifidus comprises variable fascicle lengths spanning two to five motion segments arranged predictably with the shortest fascicles attaching deepest to the spinous process [9]. Biomechanically, where structure typically dictates function, fascicles of different length will respond variably to pathology [10, 27]. We speculate that our results indicate a susceptibility of the deepest multifidus fascicles to FI and may be a result of unloading or inactivity in response to symptomatic IVD degeneration. Causation models require further study in order to optimize individualized treatments.

While our results show an association between cLBP symptoms and increased FI in the intermediate and superficial psoas, prior studies have not found such an association. The relationship between psoas FI and cLBP is inconclusive. In part, this may be a result of the relatively low levels of FI in the psoas. Unlike the multifidus and the erector spinae, we found little variation in FI levels across the distribution. As the spatial differences in the psoas are much smaller, there is more ambiguity as to potential underlying mechanisms, and the differences in FI may be more affected by confounders such as age, sex, BMI, or activity levels.

Our study limitations should be considered in wider interpretation. We limited our analysis to a single spinal level (L4L5) that, while prevalent in IVD pathology, may limit generalizability to other lumbar levels where different sizes and orientation to the CoR may influence results. With respect to confounding factors, studies have shown increased fat infiltration with age in muscles of the extremities and the trunk, including the paraspinal muscles, which may be independent of sarcopenia and muscle mass changes [28]. Further, age, sex, and intervertebral disc degeneration were shown to individually correlate with the level of fat infiltration in the combined erector spinae and multifidus, but not in the psoas [29]. To reduce the confounding effect of age and sex, the patient and control groups were age- and sex-matched. However, this results in an important limitation in that we could not study the effect that age and sex may have on the spatial distribution of FI. Future work should aim to address this gap in knowledge. Additionally, it is difficult to recruit an asymptomatic cohort for advanced sequence MRI, which is reflected in our smaller control group. The smaller sample size of both patients and controls is a limitation of this study. However, our novel findings of differential paraspinal muscle FI in cLBP may already have clinically meaningful implications and lay down an innovative foundation for future investigations.

In conclusion, our results suggest that: 1) spatial distribution patterns of FI in the PSMs differ with respect to symptoms and adjacent degenerative IVD pathology; and 2) that the deep region of the multifidus has the highest amount of FI in relation to symptomatic IVD degeneration. We also show that spatial distribution patterns of FI in the PSMs provide higher granularity and thus serve as a better diagnostic imaging-based biomarker compared to conventional summary measures for overall muscle FI%. Based on these results, we theorize that the presence of symptomatic degenerative disc pathology results in arthrogenic inhibition and a selective shutting off of the local stabilizing muscles, notably the deep multifidus, as a protection mechanism, resulting in disuse and eventually, elevated levels of fat infiltration localized to the shorter, deeper fascicles of the multifidus. Future study should look to collect longitudinal data to further explore causation. With regard to the multifactorial and uncertain nature of diagnosing patients with non-specific cLBP, establishing mechanisms of how paraspinal muscle FI occurs and understanding its relationship to painful symptoms and degenerative IVD pathology will guide the development of effective conservative therapies targeting paraspinal muscle health.

## Supplementary Information

Below is the link to the electronic supplementary material.

Supplementary file1 (DOCX 29 kb)
