# Supplementary material for: Spatial distribution of fat infiltration within the paraspinal muscles: implications for chronic low back pain
Source: Eur Spine J. 2022 Jul 1;31(11):2875–83. doi: 10.1007/s00586-022-07296-7 (PMC9637053; doi:10.1007/s00586-022-07296-7)
Supplement: Supplementary file 1 — Supplementary file1 (DOCX 29 kb) [file 586_2022_7296_MOESM1_ESM.docx]

|  | **Patients** | | **Controls** | | **Patients v. Controls** |
| --- | --- | --- | --- | --- | --- |
|  | **Mean** | **Std** | **Mean** | **Std** | **p-value** |
| **AGE** | 48.800 | 12.195 | 43.529 | 12.704 | 0.146 |
| **CEP** | 0.622 | 0.492 | 0.421 | 0.507 | 0.158 |
| **BMI** | 25.615 | 5.097 | 23.800 | 4.127 | 0.224 |
| **MC** | 0.300 | 0.464 | ~ | ~ | ~ |
| **ODI** | 34.250 | 14.144 | ~ | ~ | ~ |
| **PG** | 2.875 | 1.067 | 1.857 | 1.108 | 0.001 |
| **VAS** | 6.625 | 1.750 | ~ | ~ | ~ |
| **nT1p** | 50.602 | 14.568 | 63.823 | 24.119 | 0.011 |
| **MF FI** | 22.674 | 9.478 | 17.820 | 7.929 | 0.005 |
| **ES FI** | 22.389 | 10.796 | 21.512 | 11.777 | 0.680 |
| **Psoas FI** | 8.216 | 4.660 | 5.929 | 2.392 | 0.004 |

**Table 1: Demographics, patient-reported Outcomes, degenerative IVD pathology, and overall mean muscle FI between patients and controls** Summary statistics (mean and standard deviation) and unpaired t-test results for comparing patients and controls. nT1p: mean pixel intensity across the nucleus derived from T1p images; MF FI: overall mean FI for the MF; ES FI: overall mean FI for the ES; Psoas FI: overall mean FI for the psoas.

|  | **Patients w/ CEP n=46** | | **Patients w/out CEP n=28** | | **Controls w/ CEP n=16** | | **Controls w/out CEP n=22** | | **Patients w/ CEP v. Patients w/out CEP** | **Patients w/ CEP v. Controls w/out CEP** | **Patients w/ CEP v. Controls w/ CEP** | **Patients w/out CEP v. Controls w/out CEP** |
| --- | --- | --- | --- | --- | --- | --- | --- | --- | --- | --- | --- | --- |
|  | **Mean** | **Std** | **Mean** | **Std** | **Mean** | **Std** | **Mean** | **Std** | **p-values** | | | |
| **AGE** | 48.348 | 10.577 | 46.429 | 14.113 | 37.800 | 13.424 | 46.600 | 13.167 | 0.640 | 0.688 | 0.064 | 0.976 |
| **CEP** | 1.000 | 0.000 | 0.000 | 0.000 | 1.000 | 0.000 | 0.000 | 0.000 | 0.000 | 0.000 | ~ | ~ |
| **BMI** | 25.478 | 4.461 | 24.462 | 4.807 | 22.000 | 2.309 | 25.444 | 4.391 | 0.527 | 0.985 | 0.144 | 0.631 |
| **MC** | 0.391 | 0.499 | 0.071 | 0.267 | 0.000 | 0.000 | 0.000 | 0.000 | 0.034 | 0.015 | 0.036 | 0.387 |
| **ODI** | 35.391 | 14.503 | 31.286 | 12.976 | 0.000 | 0.000 | 0.000 | 0.000 | 0.391 | 0.000 | 0.000 | 0.000 |
| **PG** | 2.870 | 1.140 | 2.643 | 0.842 | 1.875 | 1.246 | 1.818 | 1.079 | 0.524 | 0.015 | 0.047 | 0.042 |
| **VAS** | 7.000 | 1.706 | 6.000 | 1.797 | 0.000 | 0.000 | 0.000 | 0.000 | 0.099 | 0.000 | 0.000 | 0.000 |
| **nT1p** | 48.231 | 12.348 | 53.849 | 18.509 | 69.990 | 32.080 | 64.751 | 16.493 | 0.275 | 0.003 | 0.011 | 0.139 |
| **MF FI** | 23.939 | 8.408 | 17.684 | 7.366 | 15.748 | 7.587 | 18.555 | 8.129 | 0.002 | 0.015 | 0.001 | 0.693 |
| **ES FI** | 24.285 | 10.246 | 15.574 | 6.172 | 19.573 | 12.078 | 22.257 | 12.363 | 0.000 | 0.478 | 0.136 | 0.016 |
| **Psoas FI** | 8.444 | 3.075 | 6.310 | 1.435 | 5.057 | 2.655 | 6.626 | 2.173 | 0.001 | 0.015 | 0.000 | 0.540 |

**Table 2: Demographics, patient-reported outcomes, degenerative IVD pathology, and overall mean muscle FI between patients and controls stratified by presence of CEP damage** nT1p: mean pixel intensity across the nucleus derived from T1p images; MF FI: overall mean FI for the MF; ES FI: overall mean FI for the ES; Psoas FI: overall mean FI for the psoas; n: sample size (two muscles, L/R, per subject were included for FI analyses)

|  | **Patients w/ High PG n=22** | | **Patients w/ Low PG n=58** | | **Controls w/ High PG n=4** | | **Controls w/ Low PG n=38** | | **Patients w/ High PG v. Patients w/ Low PG** | **Patients w/ High PG v. Controls w/ Low PG** | **Patients w/ High PG v. Controls w/ High PG** | **Patients w/ High PG v. Patients w/ Low PG** |
| --- | --- | --- | --- | --- | --- | --- | --- | --- | --- | --- | --- | --- |
|  | **Mean** | **Std** | **Mean** | **Std** | **Mean** | **Std** | **Mean** | **Std** | **p-values** | | | |
| **AGE** | 48.273 | 15.041 | 49.000 | 11.235 | 63.000 | 0.000 | 42.313 | 12.054 | 0.869 | 0.264 | 0.371 | 0.069 |
| **CEP** | 0.778 | 0.441 | 0.571 | 0.504 | 0.500 | 0.707 | 0.412 | 0.507 | 0.280 | 0.080 | 0.476 | 0.310 |
| **BMI** | 27.091 | 5.804 | 25.036 | 4.780 | 27.000 | 0.000 | 23.571 | 4.183 | 0.263 | 0.091 | 0.988 | 0.336 |
| **MC** | 0.455 | 0.522 | 0.241 | 0.435 | 0.000 | 0.000 | 0.000 | 0.000 | 0.198 | 0.001 | 0.260 | 0.020 |
| **ODI** | 34.182 | 11.223 | 34.276 | 15.285 | 0.000 | 0.000 | 0.000 | 0.000 | 0.985 | 0.000 | 0.015 | 0.000 |
| **PG** | 4.182 | 0.405 | 2.379 | 0.775 | 4.000 | 0.000 | 1.632 | 0.895 | 0.000 | 0.000 | 0.552 | 0.004 |
| **VAS** | 6.636 | 1.690 | 6.621 | 1.801 | 0.000 | 0.000 | 0.000 | 0.000 | 0.980 | 0.000 | 0.004 | 0.000 |
| **nT1p** | 46.332 | 7.673 | 52.222 | 16.270 | 48.500 | 9.192 | 65.526 | 24.790 | 0.259 | 0.020 | 0.725 | 0.031 |
| **MF FI** | 29.867 | 8.366 | 19.945 | 8.436 | 29.316 | 6.901 | 16.610 | 7.072 | 0.000 | 0.000 | 0.903 | 0.047 |
| **ES FI** | 29.102 | 11.708 | 19.843 | 9.331 | 44.593 | 7.823 | 19.082 | 9.224 | 0.000 | 0.001 | 0.019 | 0.696 |
| **Psoas FI** | 9.292 | 3.323 | 7.808 | 5.042 | 8.365 | 2.327 | 5.673 | 2.279 | 0.205 | 0.000 | 0.601 | 0.016 |

**Table 3 Demographics, patient-reported outcomes, degenerative IVD pathology, and overall mean muscle FI between patients and controls stratified by high and low Pfirmann Grade (PG)** Summary statistics (mean and standard deviation) and unpaired t-test results for comparing patients and controls. nT1p: mean pixel intensity across the nucleus derived from T1p images; MF FI: overall mean FI for the MF; ES FI: overall mean FI for the ES; Psoas FI: overall mean FI for the psoas, n: sample size (two muscles, L/R, per subject were included for FI analyses).

|  | **Patients w/ MC n=24** | | **Patients w/out MC n=56** | | **Controls w/ MC n=0** | | **Controls w/out MC n=42** | | **Patients w/ MC v. Patients w/out MC** | **Patients w/ MC v. Controls w/out MC** | **Patients w/ MC v. Controls w/ MC** | **Patients w/out MC v. Controls w/out MC** |
| --- | --- | --- | --- | --- | --- | --- | --- | --- | --- | --- | --- | --- |
|  | **Mean** | **Std** | **Mean** | **Std** | **Mean** | **Std** | **Mean** | **Std** | **p-values** | | | |
| **AGE** | 49.583 | 12.873 | 48.464 | 12.121 | ~ | ~ | 43.529 | 12.704 | 0.794 | 0.220 | ~ | 0.200 |
| **CEP** | 0.900 | 0.316 | 0.519 | 0.509 | ~ | ~ | 0.421 | 0.507 | 0.034 | 0.012 | ~ | 0.525 |
| **BMI** | 27.417 | 6.501 | 24.815 | 4.234 | ~ | ~ | 23.800 | 4.127 | 0.143 | 0.091 | ~ | 0.457 |
| **MC** | 1.000 | 0.000 | 0.000 | 0.000 | ~ | ~ | 0.000 | 0.000 | 0.000 | 0.000 | ~ | ~ |
| **ODI** | 38.833 | 13.442 | 32.286 | 14.212 | ~ | ~ | 0.000 | 0.000 | 0.183 | 0.000 | ~ | 0.000 |
| **PG** | 3.500 | 0.674 | 2.607 | 1.100 | ~ | ~ | 1.857 | 1.108 | 0.013 | 0.000 | ~ | 0.023 |
| **VAS** | 7.000 | 1.537 | 6.464 | 1.836 | ~ | ~ | 0.000 | 0.000 | 0.382 | 0.000 | ~ | 0.000 |
| **nT1p** | 43.457 | 5.226 | 53.664 | 16.218 | ~ | ~ | 63.823 | 24.119 | 0.041 | 0.008 | ~ | 0.087 |
| **MF FI** | 24.707 | 10.300 | 21.802 | 9.060 | ~ | ~ | 17.820 | 7.929 | 0.211 | 0.003 | ~ | 0.025 |
| **ES FI** | 22.930 | 9.975 | 22.157 | 11.209 | ~ | ~ | 21.512 | 11.777 | 0.771 | 0.621 | ~ | 0.783 |
| **Psoas FI** | 9.666 | 6.906 | 7.595 | 3.154 | ~ | ~ | 5.929 | 2.392 | 0.068 | 0.002 | ~ | 0.005 |

**Table 4 Demographics, patient-reported outcomes, degenerative IVD pathology, and overall mean muscle FI between patients and controls stratified by presence of Modic change** Summary statistics (mean and standard deviation) and unpaired t-test results for comparing patients and controls. nT1p: mean pixel intensity across the nucleus derived from T1p images; MF FI: overall mean FI for the MF; ES FI: overall mean FI for the ES; Psoas FI: overall mean FI for the psoas, n: sample size (two muscles, L/R, per subject were included for FI analyses).
